# Draft genome of *Chryseobacterium* sp. L12 isolated from mosquito habitat

**DOI:** 10.1128/mra.00787-26

**Published:** 2026-10-19

**Authors:** Kristin Gordon, Andres Salgado, Edward D. Walker, Shicheng Chen

**Affiliations:** 1 Medical Laboratory Sciences, College of Health and Human Sciences, Northern Illinois University 2848, DeKalb, Illinois, USA; 2 Department of Biology, Northern Illinois University 2848, DeKalb, Illinois, USA; 3 Department of Microbiology, Genetics and Immunology, Michigan State University 3078, East Lansing, Michigan, USA; Wellesley College, Wellesley, Massachusetts, USA

**Keywords:** *Chryseobacterium* sp. L12, mosquito, genome analysis, degradation

## Abstract

We report the draft genome of *Chryseobacterium* sp. L12, isolated from larval mosquito habitat. Its draft genome comprises 3,793,121 bp with a G + C content of 35.1% and encodes 3,501 predicted protein-coding sequences.

## ANNOUNCEMENT

Mosquito-associated *Chryseobacterium* species have been reported to contribute to larval development and participate in the degradation of organic matter (1). However, the molecular mechanisms underlying these functions remain largely unknown. Beyond their ecological significance, *Chryseobacterium* species are also recognized as opportunistic pathogens for immunocompromised patients, highlighting their potential clinical relevance (2–4).

Strain L12 was isolated from the surface of leaves collected from a native mosquito larval habitat located on Michigan State University (MSU) campus (42.708° N, 84.475° W). Leaf samples were briefly washed with 0.9% sterile saline, cut into small pieces, and further minced into fine particles in microcentrifuge tubes using sterile plastic pestles. The sample was next enriched in Casitone yeast extract (CYE) broth (10 g/L casitone, 5 g/L yeast extract, and 8 mM MgSO₄ in 10 mM Tris buffer, pH 7.6), plated onto CYE agar, and aerobically incubated at 25°C for 48 h (5–7). One yellow colony was selected and repeatedly streaked onto fresh CYE agar plates under the same incubation conditions used for enrichment until a pure culture was obtained. Culture purity was confirmed by consistent colony morphology following repeated subculturing and by microscopic examination of Gram-stained cells. Genomic DNA of strain L12 was extracted using the Wizard Genomic DNA Purification Kit (Promega, Madison, WI, USA). Sequencing libraries were prepared using the Illumina TruSeq Nano DNA Library Preparation Kit (Illumina, San Diego, CA, USA) according to the manufacturer’s instructions. Whole-genome sequencing was performed at the Research Technology Support Facility at MSU, using the Illumina MiSeq platform. Paired-end reads (2 × 250 bp) were generated with a MiSeq Reagent Kit v2 (500-cycle). Illumina sequencing generated 2,116,348 reads and 529.1 Mb of sequence data, corresponding to an estimated sequencing depth of approximately 140-fold. Raw sequencing reads were quality-filtered and *de novo* assembled using the CLC Microbial Genomics Module v25.1.1 in CLC Genomics Workbench v25.0.2 (QIAGEN Digital Insights, Aarhus, Denmark) with default parameters.

The draft genome of strain L12 comprised 46 contigs, with an *N*_50_ value of 129,267 bp. The assembled genome was 3,793,121 bp in length and had a G + C content of 35.1%. Genome annotation was performed using the National Center for Biotechnology Information (NCBI) Prokaryotic Genome Annotation Pipeline v6.10 (8). No plasmid sequences were detected using PlasmidFinder (v2.1) (https://cge.food.dtu.dk/services/PlasmidFinder/). Genome quality was assessed using CheckM (v1.0.18), revealing 98% genome completeness and no detectable contamination (9). A total of 3,588 genes were predicted, including 3,501 protein-coding genes, 20 pseudogenes, 61 transfer RNA (tRNA) genes, 3 ribosomal RNA (rRNA) genes, and 3 non-coding RNA (ncRNA) genes (Table 1). GTDB-Tk v2.3.2 (10) placed strain L12 within the *Chryseobacterium binzhouense* strain lm2^T^ species-level cluster, with 97.0% average nucleotide identity to *C. binzhouense* lm2^T^ (GenBank accession no. GCA_007474545.1) (11). The isolate is therefore reported here using its current NCBI designation, *Chryseobacterium* sp. L12.

**TABLE 1:** Genome features of *C. binzhouense* strain L12

Characteristic	Values
Genome length (bp)	3,793,121
Contigs	46
GC content (%)	35.1
Total genes	3,588
Protein-coding genes	3,501
Total RNA genes	67
rRNA genes	3
tRNA genes	61
ncRNA	3
Pseudogenes	20
*N*_50_ (bp)	129,267
*L*_50_	9
Average fold coverage (×)	50
NCBI Sequence Read Archive number	SRR39352473
NCBI whole-genome sequencing accession number	JBVYOF000000000
NCBI BioProject accession number	PRJNA1434032
NCBI BioSample number	SAMN56386495

Functional annotation using the RAST server (12) identified numerous genes associated with polysaccharide degradation. Genes involved in amino acid metabolism, protein metabolism, membrane transport, and cellular respiration were also well represented. In addition, the genome encoded proteins involved in heme utilization and iron acquisition. These genomic features are consistent with the ecological roles previously proposed for mosquito-associated *Chryseobacterium* species and provide a foundation for future genomic studies.

## Data Availability

The accession number of the assembled genome of the *Chryseobacterium* sp. L12 strain is JBVYOF000000000. Raw sequencing data have been deposited in the Sequence Read Archive under accession number SRR39352473. The BioProject designation for this project is accession number PRJNA1434032. The BioSample accession number is SAMN56386495.

## References

[1] Scolari F, Casiraghi M, Bonizzoni M. 2019. *Aedes* spp. and their microbiota: a review. Front Microbiol 10. doi:10.3389/fmicb.2019.02036 PMC6738348 31551973

[2] Lo H-H, Chang S-M. 2014. Identification, characterization, and biofilm formation of clinical *Chryseobacterium gleum* isolates. Diagn Microbiol Infect Dis 79:298–302. doi:10.1016/j.diagmicrobio.2014.01.027 24796989

[3] Klimkaitė L, Ragaišis I, Krasauskas R, Ružauskas M, Sužiedėlienė E, Armalytė J. 2023. Novel antibiotic resistance genes identified by functional gene library screening in *Stenotrophomonas maltophilia* and *Chryseobacterium* spp. Bacteria of soil origin. Int J Mol Sci 24:6037. doi:10.3390/ijms24076037 PMC10094639 37047008

[4] Chakrabarti A, Bhattacharya S, Dutta RP, Majumdar T. 2022. Blood stream infection caused by carbapenem-resistant *Chryseobacterium indologenes* harboring blaNDM-1 gene isolated from a tertiary care hospital in Tripura: an emerging threat. J Assoc Physicians India 70:11–12. doi:10.5005/japi-11001-0138 37355948

[5] Chen S, Bagdasarian M, Walker ED. 2015. *Elizabethkingia anophelis*: molecular manipulation and interactions with mosquito hosts. Appl Environ Microbiol 81:2233–2243. doi:10.1128/AEM.03733-14 PMC4345385 25595771

[6] Chen S, Kaufman MG, Korir ML, Walker ED. 2014. Ingestibility, digestibility, and engineered biological control potential of *Flavobacterium hibernum*, isolated from larval mosquito habitats. Appl Environ Microbiol 80:1150–1158. doi:10.1128/AEM.03319-13 PMC3911232 24296502

[7] Xu Y, Chen S, Kaufman MG, Maknojia S, Bagdasarian M, Walker ED. 2008. Bacterial community structure in tree hole habitats of *Ochlerotatus triseriatus*: influences of larval feeding. J Am Mosq Control Assoc 24:219–227. doi:10.2987/5666.1 PMC4106294 18666529

[8] Tatusova T, DiCuccio M, Badretdin A, Chetvernin V, Nawrocki EP, Zaslavsky L, Lomsadze A, Pruitt KD, Borodovsky M, Ostell J. 2016. NCBI prokaryotic genome annotation pipeline. Nucleic Acids Res 44:6614–6624. doi:10.1093/nar/gkw569 PMC5001611 27342282

[9] Parks DH, Imelfort M, Skennerton CT, Hugenholtz P, Tyson GW. 2015. CheckM: assessing the quality of microbial genomes recovered from isolates, single cells, and metagenomes. Genome Res 25:1043–1055. doi:10.1101/gr.186072.114 PMC4484387 25977477

[10] Chaumeil P-A, Mussig AJ, Hugenholtz P, Parks DH. 2022. GTDB-Tk v2: memory friendly classification with the genome taxonomy database. Bioinformatics 38:5315–5316. doi:10.1093/bioinformatics/btac672 PMC9710552 36218463

[11] Meng D, Liu Y-L, Li R-R, Gu P-F, Fan X-Y, Huang Z-S, Du Z-J, Li Q. 2020. *Chryseobacterium binzhouense* sp. nov., isolated from activated sludge. Int J Syst Evol Microbiol 70:618–623. doi:10.1099/ijsem.0.003800 31661045

[12] Aziz RK, Bartels D, Best AA, DeJongh M, Disz T, Edwards RA, Formsma K, Gerdes S, Glass EM, Kubal M, et al.. 2008. The RAST Server: rapid annotations using subsystems technology. BMC Genomics 9:75. doi:10.1186/1471-2164-9-75 PMC2265698 18261238

